# Biochar increases tobacco yield by promoting root growth based on a three-year field application

**DOI:** 10.1038/s41598-021-01426-9

**Published:** 2021-11-09

**Authors:** Tianbao Ren, Huanhuan Wang, Ye Yuan, Huilin Feng, Bo Wang, Gang Kuang, Yuewei Wei, Weikai Gao, Hongzhi Shi, Guoshun Liu

**Affiliations:** 1 Tobacco College of Henan Agricultural University, Zhengzhou, 450002 China; 2 Henan Biochar Engineering Technology Research Center, Zhengzhou, 450002 China; 3 Henan Biochar Technology Engineering Laboratory, Zhengzhou, 450002 China; 4 Mudanjiang Tobacco Scientific Research Institute, Herbin, 150090 China; 5 Mudanjiang Tobacco Company, Mudanjiang, 157000 China; 6 Guangdong China Tobacco Industry Co., Ltd, Guangzhou, 510032 China

**Keywords:** Plant sciences, Developmental biology

## Abstract

In order to explore the effects of biochar on root system and growth characteristics of flue-tobacco, three years of field experiments were conducted to study the influence of different biochar application levels [600 (T_1_), 1200 (T_2_), 1800(T_3_), 2400 (T_4_), 3000 (T_5_) kg/ha] and no fertilizer (CK) on the root physiological indexes and growth index of tobacco. Compared with local conventional fertilization, the application rate of N fertilizer in each treatment (except for control) was reduced by 40% to analyze the effects of different amount of biochar on the physiological indexes of tobacco roots and leaf photosynthesis during flourishing. The results showed that tobacco plants' root development status in the flourishing period was consistent with the photosynthetic physiological indexes, chlorophyll content, and leaf-area coefficient. Compared with the control, the application of biochar could increase the root vigor by 177.8%. Biochar improved the roots, increasing the total root area by 91.35% and the number of root tips by 100.9%. Meanwhile, biochar increased the net photosynthetic rate of tobacco leaves by 77.3% and the total tobacco biomass by 72.5%. Studies have shown that biochar can promote the development of tobacco roots, and then enhance the photosynthesis of leaves, so that tobacco plants can grow healthily, which is conducive to the tobacco production and the cultivation of soil.

## Introduction

Biochar is a carbon-rich solid substance produced by slow thermal cracking of biomass at high temperatures (300–1000 °C) under limited oxygen conditions^1,2^. It is a carbon-containing polymer with high carbon content, making it biochemically and thermally stable^3^. Biochar is a form of black carbon with high porosity, more negative charge, high degree of aromatization, large specific surface area, and high stability and adsorption^4,5^.

Therefore, biochar can increase soil carbon sequestration and soil conservation^6,7^. The latest biochar application method is used to mix traditional fertilizers with biochar to improve nutrient utilization and reduce agricultural investment. The acquisition of agricultural waste is simple, with low cost.

Biochar has the following functions: (1) The physico-chemical properties of the soil^8,9^ (e.g., by addressing limiting factors such as soil pH) are improved to increase crop yield and its fertilizer use^10^. (2) Biochar keeps the fertilizer efficiency in the soil, e.g., the improved soil cation exchange capacity. (3) Fertilizer utilization is improved through the fertilizer's physico-chemical properties. The unique physico-chemical properties of biochar can improve fertilizer utilization rate^11,12^. Due to its advantages, biochar, as a soil amendment, has become the focus of attention in recent years.

The application of biochar in the soil may improve the soil quality and support agricultural ecosystems^13^. For example, the positive effects of biochar include improving soil properties (i.e., reducing compaction and density; increasing pH, water holding capacity, and nutrient content)^6^, and the growth and activity of microorganisms^14^. Biochar can increase the nutrient cycling of soil and plants^15^ and optimize the structure and state of root development^16^ to increase plant productivity^17^. Changes in nutrient availability and other soil physicochemical properties caused by biochar may increase root length by 52% and root biomass by 32%^17^. Biochar absorbs nutrients around the root system and the root system absorbs abundant nutrients released from biochar^18^. In contrast, increased root length is observed in the nutrient-depleted zone^19^ due to the uptake of nutrients by biochar. It is essential to study how root traits (such as root length and biomass) respond to biochar addition, especially in heterogeneous environments such as the soil (with/without biochar areas).

At present, many problems exist in tobacco agriculture in Mudanjiang, such as too much chemical fertilizer and weak awareness of land maintenance, which has led to a significant decline in the soil fertility and unsound tobacco plant development. With low leaf maturity, insufficient oil and aroma^20,21^, a suitable soil amendment is needed to repair the local soil, solve the local tobacco-growing soil's problems, and improve the quality and efficiency of tobacco production.

The experiment (biochar localization experiment) was performed from 2015 to 2017 to improve tobacco-growing soil and plant growth, which could explore the effects of different application rates of biochar on tobacco root development and leaf photosynthetic characteristics, as well as the relationship between the coordinated growth of the overground and underground parts of tobacco. It provides a theoretical reference for further exploring the effects of long-term application of biochar or N fertilizers on crop root growth and photosynthetic characteristics, as well as the rational utilization of biomass resources and soil fertility.

## Materials and methods

### Materials

The field experiment was performed at the Tobacco Research Institute of Mudanjiang City, Heilongjiang Province, China, from April 27 to October 4, 2015, April 15 to October 8, 2016, and April 20 to October 5, 2017. The experimental site is located in the cold northeast of China (128°02′–131°18′E 43°24′–45°59'N), and has a high-latitude continental monsoon climate featuring four seasons with rain and heat in the same period. Its annual sunshine time is about 2400 h, it has an annual average temperature of 6.1 ℃ and a frost-free period of 140 d, and the annual precipitation averages about 580 mm.

The tested soil is Phaeozem (IUSS, 2014), and tobacco seedlings were transplanted on May 10, 2015, 2016, and 2017. The basic physico-chemical properties of tobacco-growing soil are as follows: soil pH of 6.9; C, N and S content of 1.371, 0.126, and 0.073%, respectively; organic matter content of 2.31%; AN content of 89%. For AN 55 mg/kg, AP is 21.587 mg/kg, and AK is 165.453 mg/kg. The biochar used in the experiment was provided by Biochar Laboratory of Tobacco College of Henan Agricultural University. Its basic physico-chemical indexes include total carbon (76.50%), moisture (9.0%), pH (8.6), TN (1.90%), TP (1.50%), and TK (0.80%).

### Methods

#### Experimental design

Six treatments were designed in this experiment, one of which was a control (CK). 600, 1,200, 1,800, 2,400 and 3,000 kg/ha biochar was applied to tobacco-growing soils, respectively (named T_1_, T_2_, T_3_, T_4_, and T_5_). After ridging the tobacco field, the fertilizer is applied between the two tobacco plants on the ridge platform. The fertilization method was hole application, and the fertilization points were 15 cm from the tobacco plant. 0.06 ha plot was used for each treatment and it was repeated three times. The row spacing was 120 cm and the plant spacing was 55 cm. Base fertilizer (conventional fertilizer) was applied in the strips of 4.5 kg/ha (60% of the normal fertilization amount), and no fertilizer was applied to the blank control. 150 kg/ha K_2_SO_4_ was applied in the topdressing stage of each biochar treatment group, and hole application was used for fertilization. After applying the base fertilizer, the field's water-holding capacity was maintained for 15 days before transplanting. The water and fertilizer management of all treatments was consistent with field management.

#### Research content and methods

Since most of the physiological indexes of tobacco plants reached their peaks on the 60th day after transplantation of tobacco plants^22^, the middle leaf (the 9th leaf from bottom to top, growing well in good light conditions), was selected to measure the photosynthetic characteristics of tobacco leaves during this period. At 9:30–11:30 on a sunny day with the light intensity greater than 1000 μmol/(m^2^·s), the experiment was repeated three times, with five plants (fixed tobacco plants) for each treatment. LI-6400 (Beijing Ligaotai Technology Co., Ltd.) portable photosynthesis instrument was used to measure the leaf net photosynthetic rate, stomatal conductance, intercellular CO_2_ concentration, and transpiration rate. After selecting the same leaf of the tobacco plant with the same photosynthetic characteristics, the length, width, and relative chlorophyll value (SPAD value) of the central leaf were determined.

The leaf area is calculated by *d* = (*L* + 2* W*)/3, where *d*, *L*, and *W* represent the leaf area, maximum leaf length, and maximum leaf width, respectively. The relative value of chlorophyll (SPAD value) was measured by SPAD-502PLUS produced by Shandong Hengmei Electronic Technology Co., Ltd. Root vigor was measured by the TTC method and root sampling. The method was as follows: take three tobacco plants for each replicate of each treatment, as well as the full roots, and store them at low temperature, and measure them in time after washing with clean water.

The root structure was measured by CI-600 produced by Shanghai Zequan Technology Co., Ltd.; the leaf area coefficient was measured by the HJ03-LAI-2200 plant canopy analyzer of Beixin Instrument Company. The obtained whole tobacco plant sample was placed at 115 °C for 25 min to inactivate the cells, then dried at 65 °C, and then weighed. The result obtained was the dry matter mass of the tobacco plant.

### Data processing

Origin 2017 was used to make histograms and line graphs, and SPSS 20.0 was used to perform the single-factor analysis of variances for different treatments. Besides, the LSD method was used for multiple comparisons.

### Ethics approval

The experimental research and field studies on plants, including the collection of plant material, complied with relevant institutional, national, and international guidelines and legislation. The appropriate permissions and/or licenses for collection of plant or seed specimens were obtained for the study.

## Results and discussions

### Effect of biochar on the physiological indexes of tobacco roots

#### Effect on root vigor

**Figure 1:**
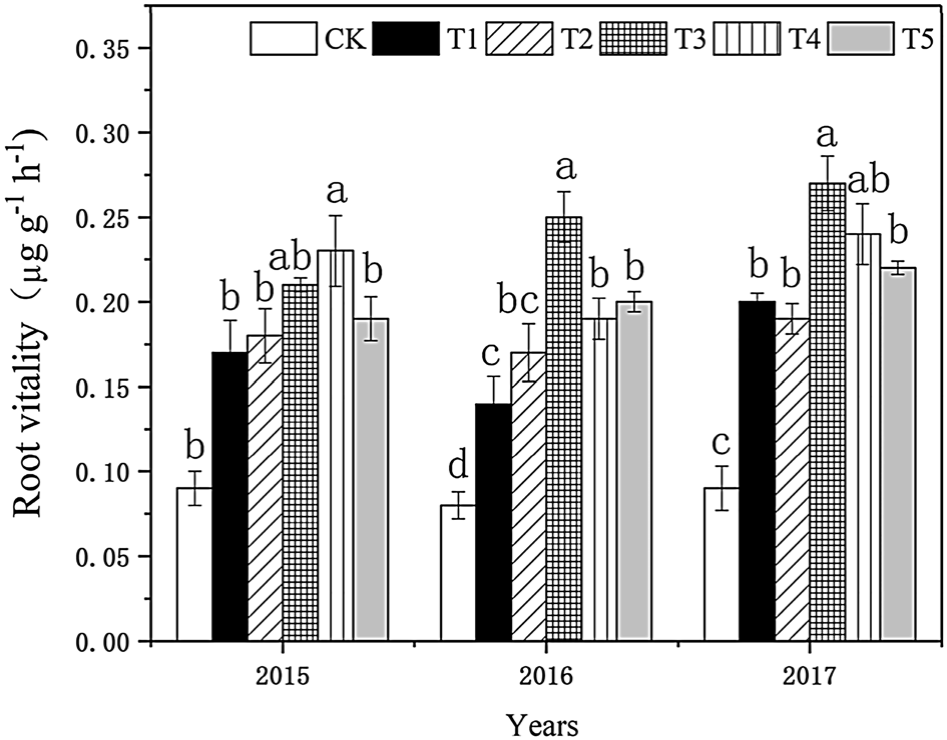
Effect of biochar treatment on the root vigor of tobacco. Note: Six biochar application levels: CK = 0 kg/ha, T1 = 600 kg/ha, T2 = 1200 kg/ha, T3 = 1800 kg/ha, T4 = 2400 kg/ha, T5 = 3000 kg/ha; The error bars are standard deviation; different letters on the error lines indicate significant differences between different treatments (α = 0.05).

As shown in Fig. 1, the changing trend of root vigor after biochar application was the same from 2015 to 2017. With the increased biochar application, the root vigor first increased and then decreased, in 2015, the root vitality reached the maximum at the T3 treatment, and in 2016–207, the root vitality reached the maximum at the T4 treatment.. There was no significant difference in root vigor among the treatments in 2015, and the difference between the biochar treatment and the control group treatment was not statistically significant. Among them, the root vigor in T_3_ and T_4_ was 110.01% and 120.03% higher than in the control, respectively. The root vigor in T_3_ was higher than in other treatments with a significant difference, while the difference among T_2_, T_4_, and T_5_ was not significant. Each treatment was significantly different from the control. T_4_ treatment had the highest root vigor, 178.02% higher than in the control. The changing trend of root vigor among treatments in 2017 was similar to that in the previous two years. However, the root vigor in biochar treatment in 2017 was higher than in the previous two years. The vitality of each line in T_3_ treatment was the highest, 164.12% higher than in the control group. The treatment of 1800 kg/ha biochar was more conducive to increasing root vigor.

#### Effect on the total surface area of the root system

**Figure 2:**
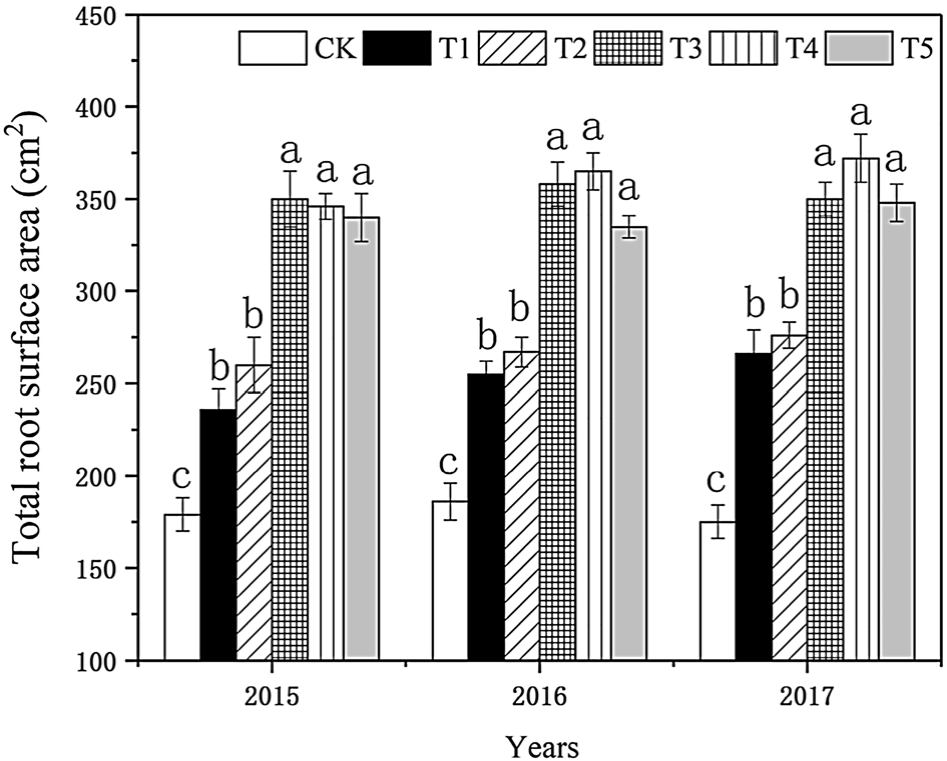
Effect of biochar treatment on total surface area of tobacco roots. Note: Six biochar application levels: CK = 0 kg/ha, T1 = 600 kg/ha, T2 = 1200 kg/ha, T3 = 1800 kg/ha, T4 = 2400 kg/ha, T5 = 3000 kg/ha; The error bars are standard deviation; different letters on the error lines indicate significant differences between different treatments (α = 0.05).

As shown in Fig. 2, the total root area in each treatment increased with increased biochar, and the total root area first increased and then decreased, consistent with the increasing trend of root vigor. In 2015, the differences between T_2_, T_3_, T_4_, T_5_, and the control reached a significant level, and the total root area in T_3_ and T_4_ was 94.00% and 91.04% larger than in the control, respectively. The differences between the treatments in 2016 were similar to those in 2015. T_4_ stood out in 2016, with the total root area 73.02% more than in the control; T_4_ had the largest total root area in 2017, 96.01% more than in the control.

The changing trend of the root system's total variable area that year was similar to that in the previous two years. On the whole, with the increased biochar application, the total surface area of the root system in each treatment showed an increasing trend. The difference among T_3_, T_4_, and T_5_ treatments was not significant, but the total surface area in these three treatments were larger than in other sources in three years. The difference between T_1_ and T_2_ was not significant, but the total surface area in T_1_ and T_2_ was larger than in the control. It showed that biochar could increase the root system's total area and help the root system absorb nutrient elements in the tobacco-growing soil. The effect of T_4_ was the most prominent.

#### Effect on the total number of root tips

**Figure 3:**
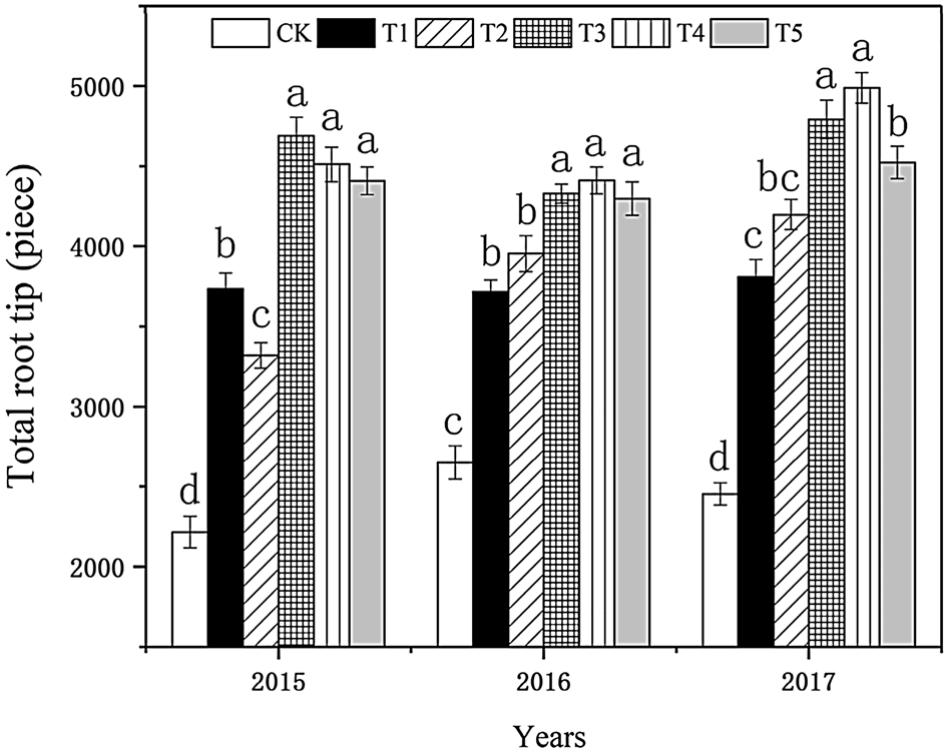
Effect of biochar treatment on the total root tips of tobacco. Note: Six biochar application levels: CK = 0 kg/ha, T1 = 600 kg/ha, T2 = 1200 kg/ha, T3 = 1800 kg/ha, T4 = 2400 kg/ha, T5 = 3000 kg/ha; The error bars are standard deviation; different letters on the error lines indicate significant differences between different treatments (α = 0.05).

Figure 3 shows the overall change of the total root tip number in each treatment. With the increased biochar, it first increased and then decreased, which was similar to the changing trend of root vigor. The difference between T_2_, T_3_, T_4_, T_5_, and the control in 2015 reached a significant level. The total number of root tips in T3 was 100.00% more than in the control. The difference between the treatments in 2016 was similar to that in 2015, and the total number of root tips in T_4_ was 69.57% more than in the control. In 2017, it was the largest in T_4_, 115.00% more than in the control. With the increased biochar application, the total number of root tips of tobacco plants showed an increasing trend. T_3_ and T_4_ treatments were more conducive to the increase of root tips.

### Effect of biochar treatment on the tobacco leaf area

As can be seen from Table 1, the area of middle leaf in each treatment was larger than in the contro l in 2015. Moreover, the difference was significant compared with the control. The changing trend of treatments was as follows: T_4_ > T_5_ > T_2_ > T_3_ > T_1_. The maximum leaf area in T_4_ was 73% more than in the control. Besides, the changing pattern of the middle leaf area in each treatment in 2016 was similar to that in 2015, with the leaf area in T_4_ 26.15% more than in the control. The changing trend of treatments in 2017 was as follows: T_4_ > T_3_ > T_5_ > T_2_ > T_1_ > the control, and the leaf area in T_4_ was 60.88% more than in the control. It showed that T_4_ had an excellent promoting effect on the opening of tobacco leaves.

**Table 1:** Effect of biochar treatment on tobacco leaf area coefficient.

Leaf area (cm^2^)	CK	T_1_	T_2_	T_3_	T_4_	T_5_
2015	457.26d	639.28c	762.39b	755.92b	806.51a	792.38a
2016	519.56c	763.38b	761.05b	807.06a	811.28a	786.34ab
2017	501.36c	778.49b	793.15	824.94a	877.52a	806.51b

Different lowercase letters indicate significant differences between treatments in the same year at the 0.05 level.

### Effect of biochar treatment on tobacco chlorophyll

As can be seen from Table 2, the SPAD value (relative value of chlorophyll) of the middle leaf in the peak period of 2015 was higher than that in the control, with a significant difference. The changes among the treatments were as follows: T_4_ > T_5_ > T_3_ > T_1_ > T_2_. The SPAD value was 57.68% higher than that in the control; the SPAD change pattern of the treatments in 2016 was similar to that in 2015, and the SPAD value in T_4_ was 50.91% higher than in the control. In 2017, the SPAD value decreased after increasing, with the increase of biochar. The SPAD value in T_4_ reached its peak, 50.47% higher than in the control. The SPAD value could directly reflect the chlorophyll content, and T_4_ could promote the accumulation of chlorophyll in tobacco.

**Table 2:** Effect of biochar treatment on tobacco chlorophyll.

SPAD	CK	T_1_	T_2_	T_3_	T_4_	T_5_
2015	29.82c	40.59b	39.56b	43.21ab	47.02a	45.36a
2016	30.23c	39.76b	39.58b	40.13ab	45.62a	41.38a
2017	37.69c	52.93a	41.21b	42.19b	49.11a	46.38a

Note: Different lowercase letters indicate significant differences between treatments in the same year at the 0.05 level. "SPAD" represents the relative value of chlorophyll content.

### Effect of biochar on leaf photosynthetic characteristics and leaf area coefficients of flue-cured tobacco

#### Effect on the photosynthetic characteristics of leaves

**Figure 4:**
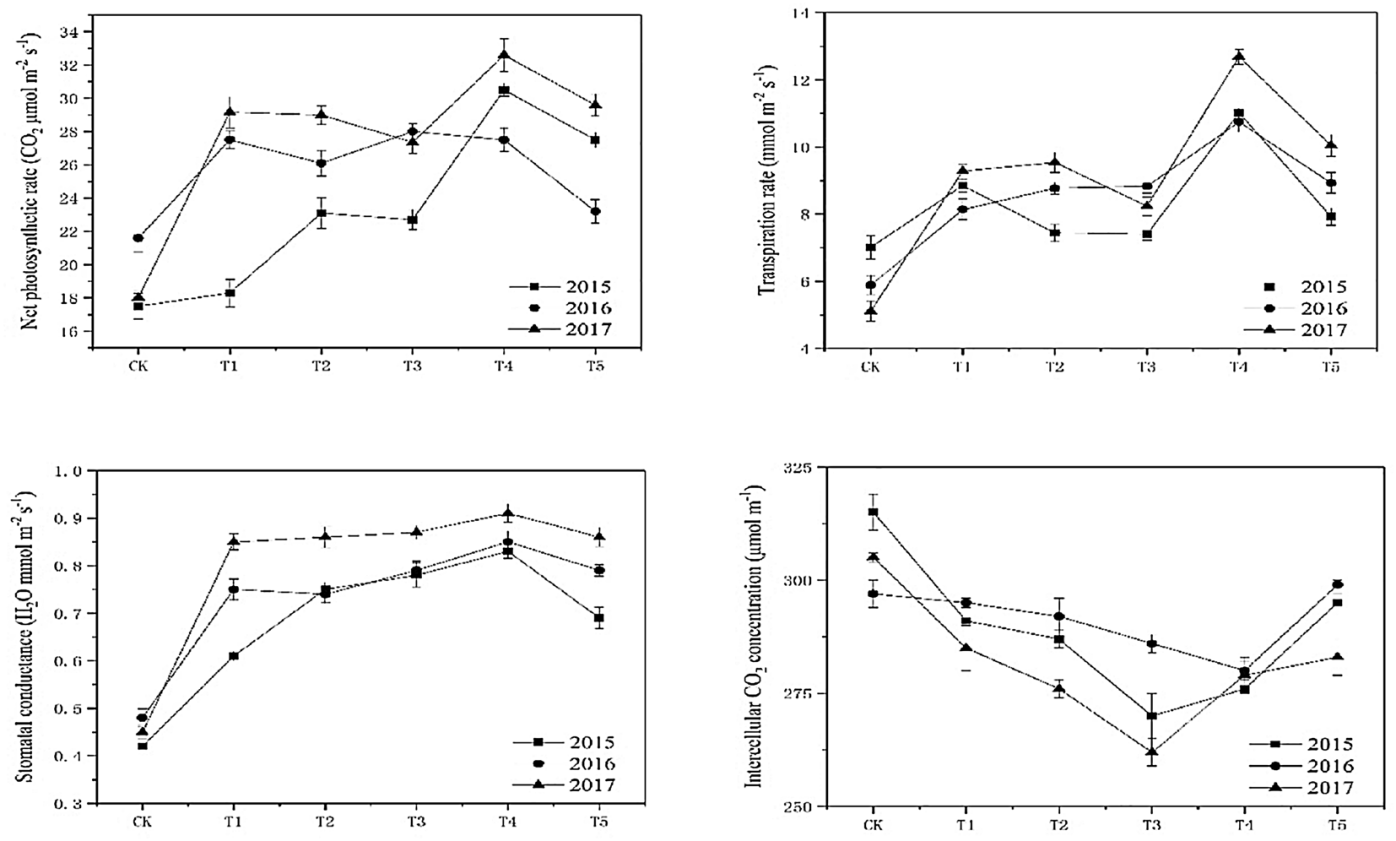
Effect of biochar treatment on the photosynthetic characteristics of tobacco. Note: Six biochar application levels: CK = 0 kg/ha, T1 = 600 kg/ha, T2 = 1200 kg/ha, T3 = 1800 kg/ha, T4 = 2400 kg/ha, T5 = 3000 kg/ha; The error bars are standard deviation.

Figure 4 shows that the net photosynthetic rates of leaves in 2015, 2016, and 2017 decreased after increasing, with the increased biochar application. In 2015, it peaked in T_3_; in 2016, it peaked in T_4_; in 2017, it peaked in T_4_. Table 1 shows that the leaf opening and SPAD value in T_3_ and T_4_ were relatively large, which increased the net photosynthetic rate. The two values were higher in biochar treatment than in the control. The changing trend of leaf transpiration rate was consistent with that of the net photosynthetic rate in each treatment, and it was higher than in the control. The three-year data peaked in T_4_.

In 2015, 2016, and 2017, the changes of leaf stomatal conductance were also consistent with those of the net photosynthetic rate. Each treatment's intercellular CO_2_ concentration decreased with the increased biochar application during the three years and then increased. The changes in photosynthetic characteristics of the treated leaves were the same, which indicated that the various indexes of photosynthetic characteristics were complementary to each other in the changes, and the changes in the data of light and leaf characteristics in the two years were the same, which showed that biochar had a positive effect on flue-cured tobacco leaves.

#### Effect on leaf area coefficient

**Figure 5:**
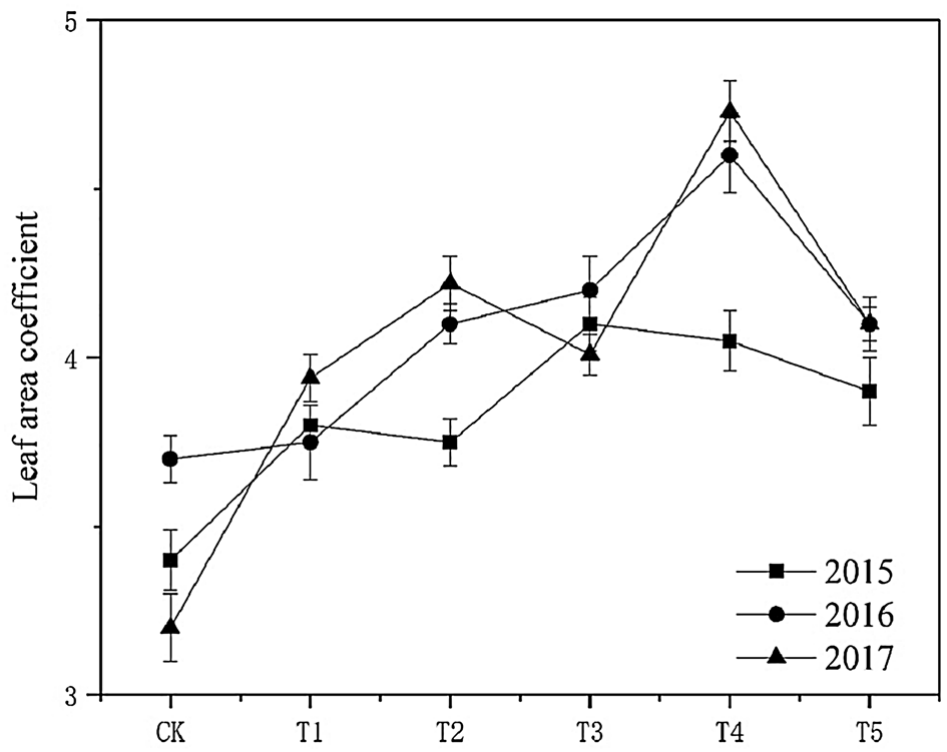
Effect of biochar treatment on tobacco leaf area coefficients. Note: Six biochar application levels: CK = 0 kg/ha, T1 = 600 kg/ha, T2 = 1200 kg/ha, T3 = 1800 kg/ha, T4 = 2400 kg/ha, T5 = 3000 kg/ha; The error bars are standard deviation.

Figure 5 shows that in 2015, 2016, and 2017, the leaf area coefficients' change remained the same. With the increase of biochar, leaf area coefficient first increased and then decreased, and reached the peak value in T4. The leaf area index in each biochar treatment was higher than in the control. The change of leaf area coefficients in each treatment was T_4_ > T_3_ > T_5_ > T_1_ > T_2_ > the control (2015); T_4_ > T_3_ > T_5_ > T_2_ > T_1_ > the control (2016); T_4_ > T_2_ > T_5_ > T_3_ > T_1_ > the control (2017). It showed that biochar could promote leaf opening and ensure the balanced nutrition of the overground part of the flue-cured tobacco plant. The effect of T_4_ was obvious.

#### Effect on the total biomass of tobacco plants

**Figure 6:**
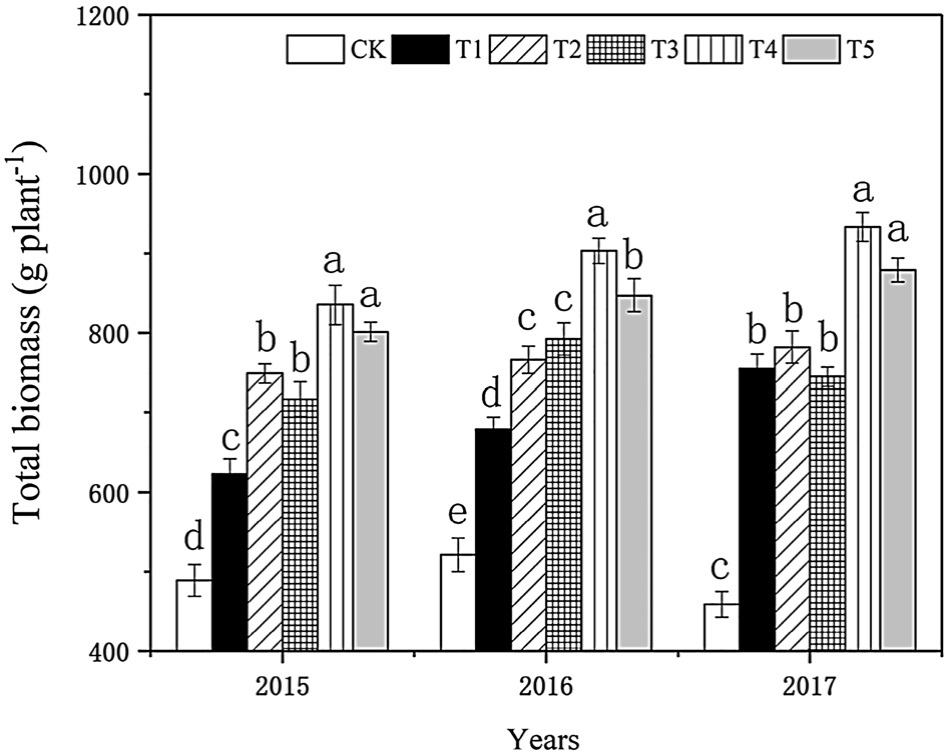
Effect of biochar treatment on total biomass of tobacco. Note: Six biochar application levels: CK = 0 kg/ha, T1 = 600 kg/ha, T2 = 1200 kg/ha, T3 = 1800 kg/ha, T4 = 2400 kg/ha, T5 = 3000 kg/ha; The error bars are standard deviation; different letters on the error lines indicate significant differences between the different treatments (α = 0.05).

Figure 6 shows that with the increase of biochar application, the total biomass in each treatment also increased. In 2015, the total biomass of tobacco plants increased with the increase of biochar application. The change was T_4_ > T_5_ > T_2_ > T_3_ > T_1_ > the control, and each biochar treatment was significantly different from the control. The changing trend of this index in 2016 was similar to that in 2015, and the index in T_4_ was 94.56% higher than in the control. The changing trend in 2017 was similar to that in the previous two years. The difference between T_3_ and T_4_ was not significant, but the total biomass of tobacco plants was higher than that in other treatments. The index in T_4_ was 117% higher than in the control. Biochar had a promoting effect on tobacco yield, and the effect of T_4_ was prominent.

## Discussions

### Effect of the amount of biochar on the root physiological indexes of flue-cured tobacco

The root system is an active absorber and synthetic organ^23^. Its growth and development status and vitality level affect the overground parts' nutritional status and yield level^24^. The vitality of the root system is an important indicator that responds to developing the root system^25,26^.With the increased biochar application rate, root vigor, total root surface area, and total root-tip number increase because biochar increases soil permeability and porosity and reduces soil bulk density. Finally, the physiological indexes of the root system are improved. When the application rate of biochar was 2400 kg/ha (T_4_), biochar had the best effect on root physiological indexes, higher than in the control. Biochar has a strong adsorption capacity, reduces the loss of nutrients in the soil, and improves the availability of nutrients in the soil, thereby improving the soil structure and utilizing nutrients by the root system. Biochar can also improve soil’s water-holding capacity and soil porosity, and soil CEC can increase by 20%^26^. Meanwhile, biochar can increase the diversity of soil microorganisms^27^. Microorganisms in soil are essential participants in material transformation^28^. When the number of microorganisms involved in the decomposition of organic carbon and nitrogen in the soil increases, the mineralization rate of carbon and nitrogen and the utilization of nutrients will increase^29^. A well-developd tobacco root system helps the growth of the overground parts, and the enhancement of leaf photosynthesis provides sufficient nutrients for root growth^30^. With the improved soil physico-chemical properties, the growth and development of roots can obtain sufficient nutrients in a suitable growth environment^31^. There are differences in the response of crops to biochar application rates. It may be affected at low application rate, but high application rate inhibits crop growth and development. This result requires further exploration of the reasons or mechanisms^32^, and this conclusion is similar to the experimental results of the work.

### Effect of biochar application on flue-cured tobacco leaf area and chlorophyll

With the increased biochar application rate, the leaf area and SPAD value of the middle leaf increased. The above two indexes in biochar treatment were higher than in the control. Among them, 2400 kg/ha of biochar treatment had apparent advantages in the above indexes because biochar could increase the respiratory metabolism rate of microorganisms^33^. The porous structure and strong adsorption properties of biochar provide a suitable habitat for soil microorganisms and have strong nutrient retention capacity^34^. Biochar can avoid the leaching of nutrient elements by reducing the dissolution and migration of water-soluble nutrient ions. It is slowly and continuously released in the soil, thereby achieving soil fertility^15,35^. It also improves the utilization of microorganisms on the substrate and improves soil fertility^36^, and the root growth. When the roots of flue-cured tobacco grow well, they can deliver sufficient water and mineral nutrients to the leaf^37^. The chlorophyll activity in the leaves is guaranteed, and the flue-cured tobacco leaves are opened in time. Previous studies show that the root system's physiological activity and the chlorophyll content of rice during the grain filling stage are significantly positively correlated^38^. This conclusion is similar to the experimental results of the work.

### Effect of biochar application on the photosynthetic characteristics, leaf area coefficients and total biomass of flue-cured tobacco in the flourishing period

Among the biochar treatments, 2400 kg/ha of biochar treatment has the most significant impact on the photosynthetic physiological indexes of flue-cured tobacco leaves because the biochar application in this treatment is more conducive to the development of leaves and the optimization of the physiological indexes of photosynthesis^39^. The change of photosynthetic characteristics is similar to the change of the root physiological index. Furthermore, the tobacco plants in 2400 kg/ha of biochar treatment have greater root vigor, total root tip number, and total root absorption area in the flourishing period, promoting the tobacco plants to maintain good photosynthetic physiological performance and chlorophyll synthesis. Biochar has advantages such as reducing the concentration of carbon dioxide between cells, and increasing net photosynthesis rate, transpiration rate, stomatal conductance, and leaf area coefficients, which may be related to the physiological characteristics of roots and chlorophyll. When the roots and leaves of flue-cured tobacco are well developed, the supply of mineral nutrients, water, and enzymes required for photosynthesis is sufficient to optimize the relevant indexes of photosynthetic characteristics and make the leaf development healthier. Moreover, the leaf area coefficients increase. The growth status of the root system of flue-cured tobacco is closely related to the leaf photosynthetic physiological status, and the physiological indexes of photosynthesis and the development of the overground parts are in connection with the yield and quality of flue-cured tobacco^40^. The addition of biochar increases the net photosynthetic rate of rice and plays a significant role in promoting rice growth during the vegetative and maturing stages^41^. This conclusion is consistent with the results of our work. The increase in leaf area index of the flue-cured tobacco lays the foundation for improving the maturity and yield quality of the flue-cured tobacco at the later stage. Biochar can increase the total biomass of tobacco, which is an important economic crop^42^. Therefore, the increased total biomass is of great significance to farmers.

## Conclusions

The application of biochar can improve the environment of tobacco root growth, root vigor, primary root morphology, flue-cured tobacco leaves, and physiological indexes. The effects of biochar on leaf photosynthetic physiological indexes, leaf area, leaf area coefficient, and chlorophyll prove that biochar’s role in promoting leaf photosynthetic characteristics results from the coordination, complementation, and synergistic development of flue-cured tobacco roots and overground parts. By changing the characteristics of the soil, biochar promotes tobacco growth and coordinates the relationship between roots and leaves, which is of great significance to the improvement of the quality of raw materials in the tobacco production process.
